# Supplementary material for: PD-L1ATTAC mice reveal the potential of depleting PD-L1 expressing cells in cancer therapy
Source: Aging (Albany NY). 2023 Mar 22;15(6):1791–807. doi: 10.18632/aging.204598 (PMC10085585; doi:10.18632/aging.204598)
Supplement: Supplementary Figures [file aging-15-204598-s002.pdf]

SUPPLEMENTARY FIGURES

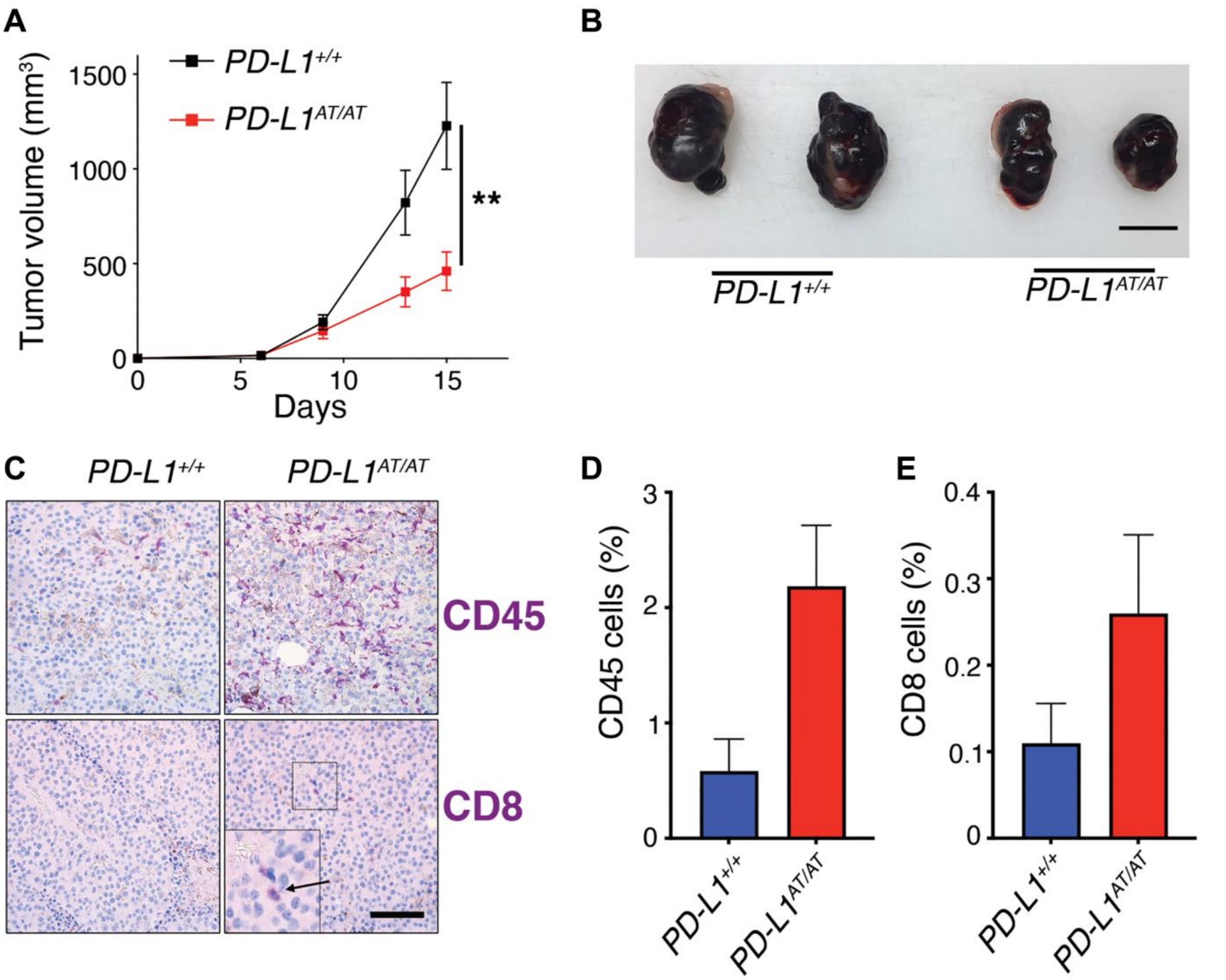

Supplementary Figure 1. Resistance to B16-F10 melanoma allografts in *PD-L1*$^{AT/AT}$ mice. (**A**) Growth of B16-F10 allografts subcutaneously implanted into the left flank of *PD-L1*$^{+/+}$ and *PD-L1*$^{AT/AT}$ mice. The *p* value was calculated with mixed-effects analysis. $^{**}p <$ 0.01 (**B**) Representative picture of the melanoma allografts from these analyses isolated at day 15. Scale bar (black) indicates 1 cm. (**C**) Immunohistochemistry of CD45 and CD8 in B16-F10 allografts isolated at day 15from of *PD-L1*$^{+/+}$ and *PD-L1*$^{AT/AT}$ mice. An inset is magnified to illustrate the presence of tumor-infiltrating CD8+ cytotoxic T cells in the allografts grown in mutant mice. Scale bar (black) indicates 100 µm. (**D**, **E**) Quantification of CD45+ (**D**) and CD8+ (**E**) cells from the analyses shown in (**C**).

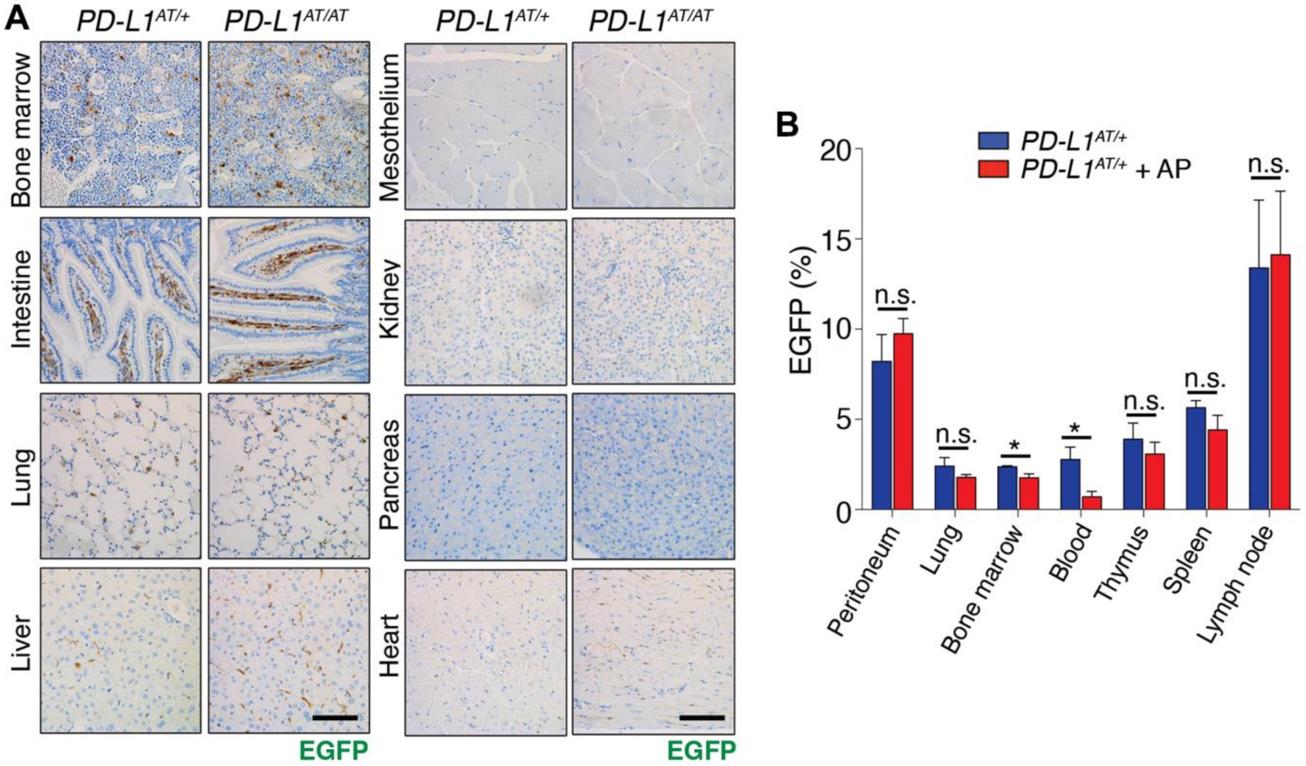

Supplementary Figure 2. Characterization of the PD-L1^{ATTAC} mouse model. (**A**) EGFP immunohistochemistry (IHC) from the bone marrow, intestine, lung, liver, mesothelium, kidney, pancreas and heart of *PD-L1*$^{AT/+}$ and *PD-L1*$^{AT/AT}$ mice. Scale bar (black) indicates 100 µm. (**B**) Percentage of EGFP+ cells as revealed by FACS in the indicated organs from control and AP-treated *PD-L1*$^{AT/+}$ mice. AP20187 (2.5 mg/kg) was administered via i.v. for three consecutive days. The *p* value was calculated with unpaired *t*-test. Abbreviation: n.s.: non-significant, $^{*}p < 0.05$.

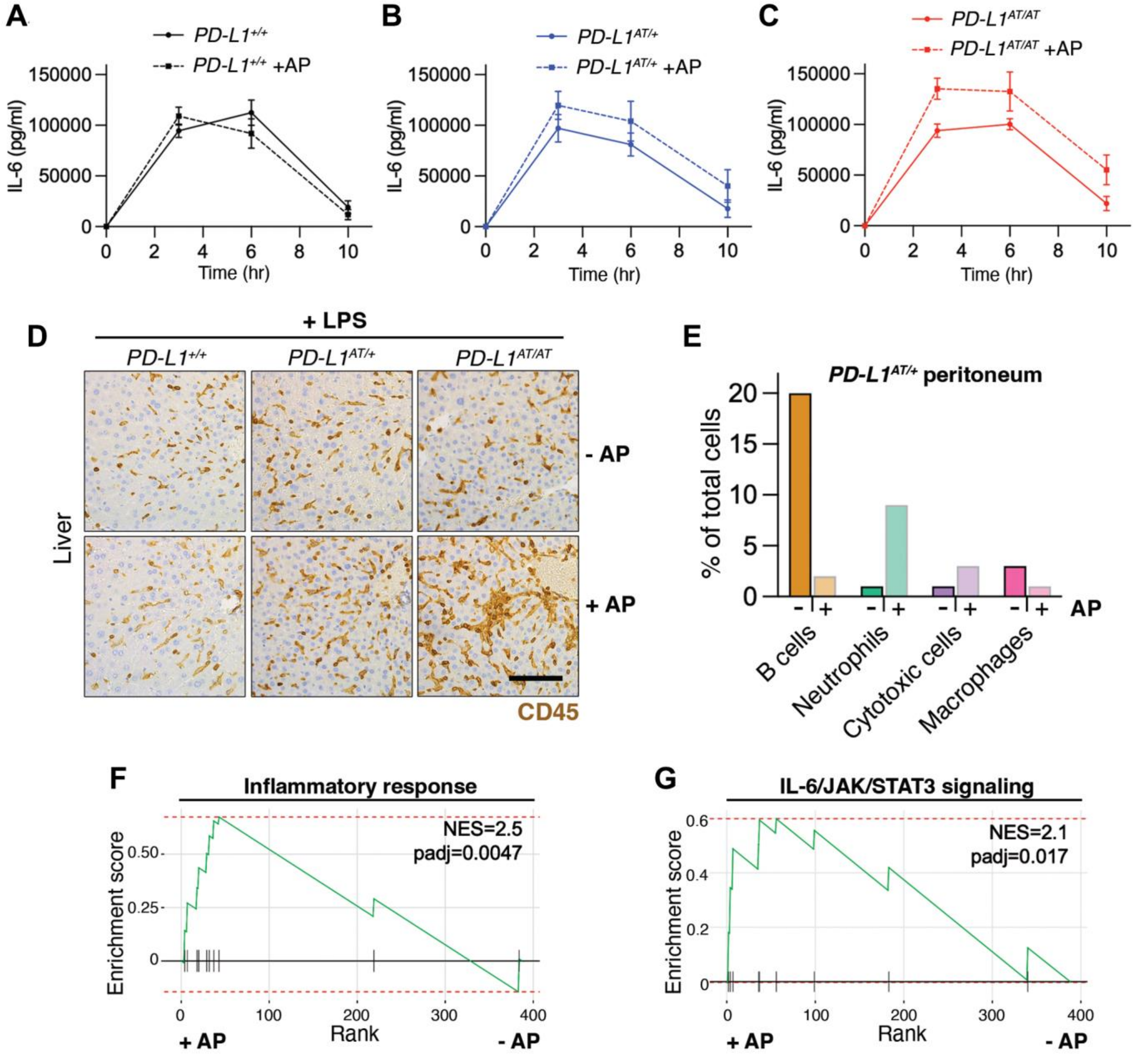

Supplementary Figure 3. Depletion of PD-L1⁺ cells sensitizes mice to LPS. (**A**–**C**) Kinetics of IL-6 accumulation and clearance in plasma isolated from *PD-L1*$^{+/+}$, *PD-L1*$^{AT/+}$ and *PD-L1*$^{AT/AT}$ mice after LPS injection as determined by ELISA. (**D**) IHC of CD45 in the livers of *PD-L1*$^{+/+}$, *PD-L1*$^{AT/+}$ and *PD-L1*$^{AT/AT}$ mice after LPS injection. Mice were treated via i.p. with AP (2.5 mg/kg) for 3 days and i.p. with 10 mg/kg LPS on the following day. Scale bar (black) indicates 100 µm. (**E**) Quantification from the single-cell sequencing analysis shown in Figure 4E, indicating the changes in the cell repertoire in the peritoneum from *PD-L1*$^{AT/+}$ mice upon AP treatment. (**F**, **G**) Preranked GSEA on the genes from the hallmarks "Inflammatory response" (**F**) and "IL-6/JAK/STAT3 signaling" (**G**) obtained from scRNAseq data comparing the transcriptomes of cytotoxic cells from *PD-L1*$^{AT/+}$ mice upon AP treatment.

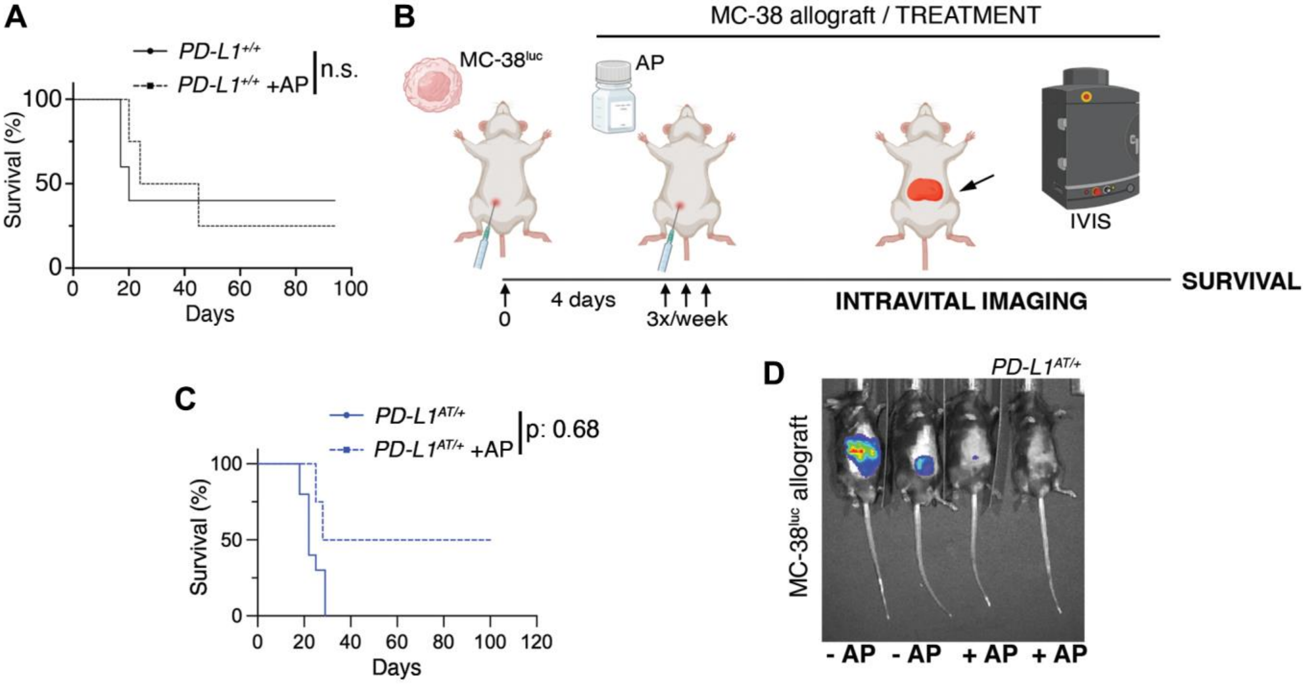

Supplementary Figure 4. Impact of depleting PD-L1^{+} cells in MC-38^{luc} allografts. (**A**) Kaplan- Meier survival curve of control and AP20187-pretreated *PD-L1*$^{+/+}$ mice after i.p. inoculation of MC-38^{luc} allografts. The *p* value was calculated with the Mantel-Coxlog rank test. Abbreviation: n.s.: non-significant. (**B**) Schematic overview of the treatment experimental workflow. 5 × 10^{5} MC-38^{luc} cells were intraperitoneally injected into mice. 4 days later mice were injected i.p. with AP20187 (2.5 mg/kg) 3 times a week for the duration of the experiment. (**C**) Kaplan-Meier survival curve of control and AP20187-pretreated *PD-L1*$^{AT/+}$ mice after i.p. inoculation of MC-38^{luc}allografts in the treatment model. The *p* value was calculated with the Mantel-Coxlog rank test. (**D**) Representative IVIS image of mice from the experiment definedin (**C**).
